# Photocatalyzed syntheses of phenanthrenes and their aza-analogues. A review

**DOI:** 10.3762/bjoc.16.123

**Published:** 2020-06-25

**Authors:** Alessandra Del Tito, Havall Othman Abdulla, Davide Ravelli, Stefano Protti, Maurizio Fagnoni

**Affiliations:** 1 Photogreen Lab, Department of Chemistry, University of Pavia, Viale Taramelli 12, 27100 Pavia, Italy; 2 Chemistry Department, College of Science, Salahaddin University, Erbil, Iraq

**Keywords:** phenanthrenes, phenanthridines, photocatalysis, radicals, visible light

## Abstract

Phenanthrenes and their aza-analogues have important applications in materials science and in medicine. Aim of this review is to collect recent reports describing their synthesis, which make use of radical cyclizations promoted by a visible light-triggered photocatalytic process.

## Introduction

Phenanthrenes are widely investigated compounds, due to the impressive number of diverse applications involving this scaffold, ranging from medicinal chemistry [1] to materials sciences, including their use in optoelectronics [2–3] and in the design of dye-sensitized solar cells (DSSC) [4]. Typical methods for the construction of a phenanthrene core involve transition-metal-catalyzed cycloisomerizations starting from arynes [5–6], *o*-alkynyl-biaryls [7–8], or substituted *N*-tosylhydrazones [9].

However, since the introduction in 1964 of the Mallory photocyclization of stilbenes [10] leading to phenanthrenes, the interest in protocols for the construction of poly(hetero)aromatic cores under photochemical conditions has increased steadily, especially when solar light may be used [11].

**Figure 1:**
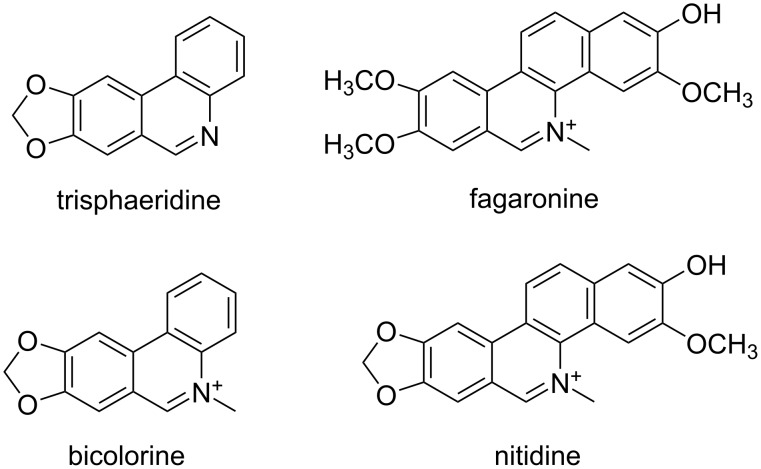
Bioactive phenanthridine and phenanthridinium derivatives.

Moreover, aza-analogues of phenanthrenes, in particular phenanthridines, are substructures present in a wide range of both natural and synthetic products, including trisphaeridine [12] (that exhibits an anti-HIV-I protease activity) and the antifungal sanguinarine [13]. Some phenantridinium derivatives are known as well, notably fagaronine (a DNA topoisomerase 1 inhibitor [14] and DNA intercalator), bicolorine (5-methyl-[1,3]dioxolo[4,5-*j*]phenanthridin-5-ium ion, a trypanocidal) [15], and the antimalarian nitidine, as well as ethidium bromide (EB), that has been employed as a DNA- and RNA-fluorescent marker for a long time (some examples are collected in Figure 1). For these reasons, apart from the well-known dehydrative ring-closure of acyl-*o*-xenylamines in the presence of phosphorus oxychloride proposed by Morgan and Walls [16], several synthetic protocols for constructing the phenanthridine structure have been reported [17–18]. These include, among the others, the anionic ring-closure of 2-cyanobiaryls by using organometallic reagents [19–20], and an impressive number of transition-metal-catalyzed C(sp^2^)–C(sp^2^) cross-coupling processes [21–23].

In the last decade, however, photochemical reactions, especially those promoted by a photocatalyst, have revolutionized the way chemists can arrive to important chemical scaffolds [24–26]. Indeed, the photocatalytic approach combines unparalleled mild conditions, due to the use of photons as traceless reagents that leave no residue behind [27–28], with the exploitation of rather inexpensive visible light (or sunlight, when possible) irradiation [29]. In general terms, photocatalysis smoothly gives access to reactive radical intermediates [30], mainly carbon-centered [31–33], or nitrogen-centered radicals [34–35]. In turn, these species have been extensively employed in radical cyclizations for the synthesis of polycondensed aromatics, with a focus on those containing heteroatoms [36–39]. The aim of the present review is to summarize the recent efforts in the design and optimization of photocatalyzed procedures for the synthesis of phenanthrenes and their nitrogen-containing heteroarene analogues via the intermediacy of a radical. However, some interesting approaches carried out under photomediated or photocatalyst-free conditions have been likewise included for the sake of completeness.

## Review

### 1 Synthesis of phenanthrenes

**Scheme 1:**
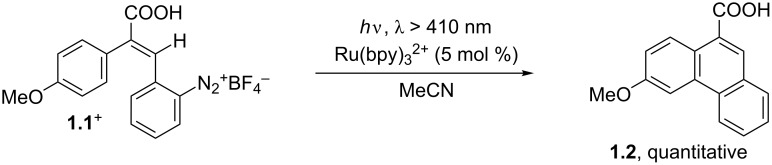
Synthesis of phenanthrenes by a photo-Pschorr reaction.

The photocatalyzed synthesis of the phenanthrene skeleton is a quite unexplored field, a notable exception being the seminal work published in 1984 by Cano-Yelo and Deronzier, where the authors reported one of the first applications of the Ru(bpy)_3_^2+^ complex in photoredox catalysis (Scheme 1). This contribution described a photo-Pschorr cyclization occurring on a stilbene diazonium salt (e.g., **1.1^+^**) with the intermediacy of an aryl radical [40].

**Scheme 2:**
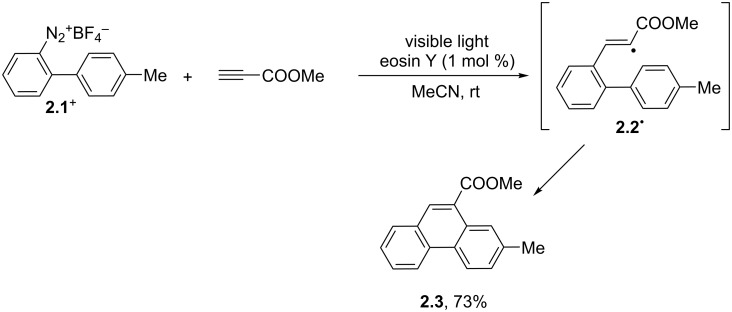
Synthesis of phenanthrenes by a benzannulation reaction.

Alternative strategies for the synthesis of phenanthrenes have been later reported, including the adoption of [4 + 2] benzannulations between biaryl derivatives and alkynes [41–42]. Scheme 2 illustrates one of such cases where an aryl radical, formed via the photocatalyzed reduction of diazonium salt **2.1^+^**, added to methyl propiolate. Ensuing cyclization of the resulting vinyl radical **2.2^·^** finally yielded the desired phenanthrene **2.3** [41].

**Scheme 3:**
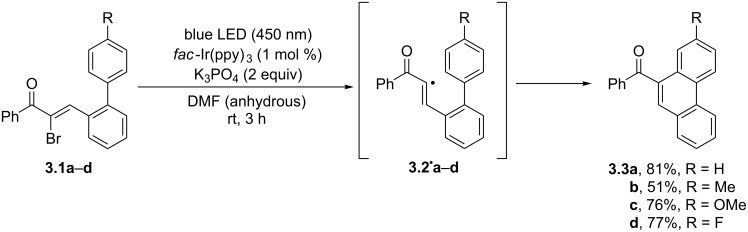
Photocatalytic cyclization of α-bromochalcones for the synthesis of phenanthrenes.

A different approach involves the intramolecular cyclization of α-bromochalcones (Scheme 3). Thus, compounds **3.1a–d** underwent a one-electron reduction by the excited photocatalyst *fac*-Ir(ppy)_3_. Upon bromide anion loss, the α-keto vinyl radicals **3.2^·^a–d** were then formed, which smoothly added onto the vicinal aromatic ring in an intramolecular fashion, affording phenanthrene derivatives **3.3a–d** upon rearomatization. Notably, the process offers a wide substrate scope and the products are obtained with complete regioselectivity [43].

### 2 Synthesis of phenanthridines or related azaarenes

**Figure 2:**
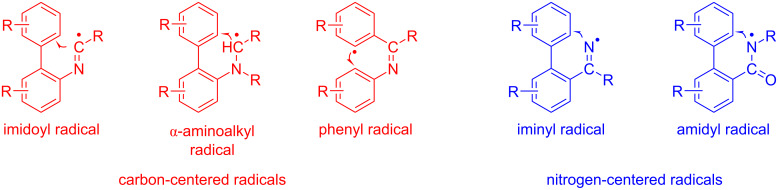
Carbon-centered and nitrogen-centered radicals used for the synthesis of phenanthridines.

Under photocatalyzed conditions, phenanthridines are mostly obtained via an intramolecular radical cyclization occurring in a biphenyl moiety or a related system containing two aromatic rings. Either carbon-centered radicals (e.g., imidoyl, α-aminoalkyl, or phenyl) or nitrogen-centered radicals (e.g., iminyl or amidyl) can be used for this purpose as shown in Figure 2. Accordingly, the azaarene may be formed by an intramolecular C–C or C–N bond-formation event, as detailed in the following.

#### 2.1 Synthesis of phenanthridines via photocatalyzed intramolecular C–C bond formation

**Scheme 4:**
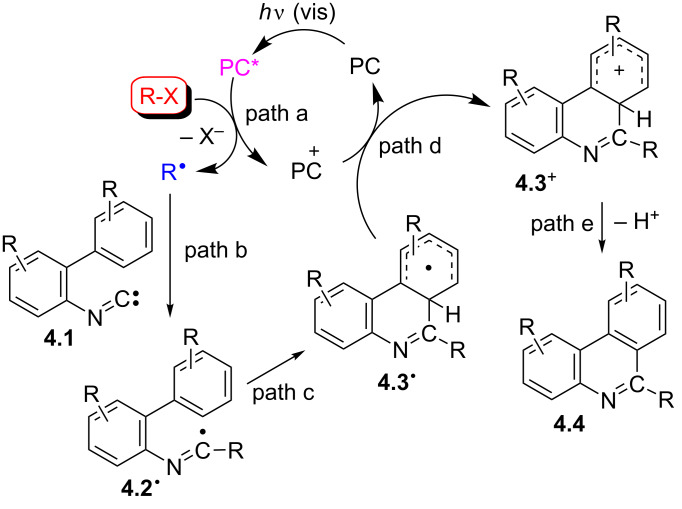
General scheme describing the synthesis of phenanthridines from isocyanides via imidoyl radicals.

A typical approach makes use of imidoyl radicals [30,44] as the key intermediates. Among the different methods proposed to construct the phenanthridine core, somophilic (radical) isocyanide addition [45–47] is probably the most adopted one, in view of the versatility and low cost of the starting substrates. Accordingly, several protocols for the synthesis under photocatalytic conditions of phenanthridines starting from 2-isocyano-1,1'-biaryls **4.1** have been reported, as summarized in Scheme 4. Along with substrate **4.1**, a radical source R–X and a photocatalyst (PC), which is activated upon visible-light irradiation, are usually required. Oxidative quenching of the photoexcited PC* by R–X (path a) affords, upon loss of the nucleofugal group X^−^, the intermediate R**^·^**, that is in turn trapped by **4.1** (path b). The resulting imidoyl radical **4.2^·^** undergoes cyclization to **4.3^·^** (path c) that is oxidized by PC**^·+^**, thus restoring the starting photocatalyst PC and forming the Wheland intermediate **4.3**^+^ (path d). Deprotonation of **4.3**^+^ (path e) finally yields the desired phenanthridine **4.4**.

**Scheme 5:**
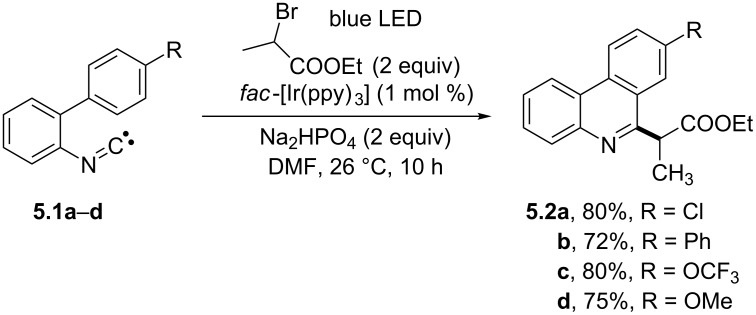
Synthesis of substituted phenanthridines involving the intermediacy of electrophilic radicals.

Different radical sources R–X have been adopted to generate carbon or heteroatom-based radicals according to the general photocatalytic strategy gathered in Scheme 4, for their use in the construction of phenanthridine scaffolds. As an example, unsubstituted alkyl radicals were easily accessed by the photocatalyzed reduction of the corresponding bromides, in turn promoting an efficient radical addition onto isonitriles. In one instance, the dimeric gold complex [Au_2_(dppm)_2_]Cl_2_ (dppm = bis(diphenylphosphino)methane) acted as the photocatalyst and activated the bromoalkanes through an oxidative quenching mechanism [48]. Phenanthridines may be also formed by the initial addition of an electrophilic radical onto isonitriles. Thus, a library of 6-alkylated phenanthridines (**5.2a**–**d** in Scheme 5) and other nitrogen-based heterocycles have been prepared from biaryls **5.1a–d** in up to excellent yields at room temperature by using α-bromoesters as radical precursors and [*fac*-Ir(ppy)_3_] as the photoredox catalyst [49].

**Scheme 6:**
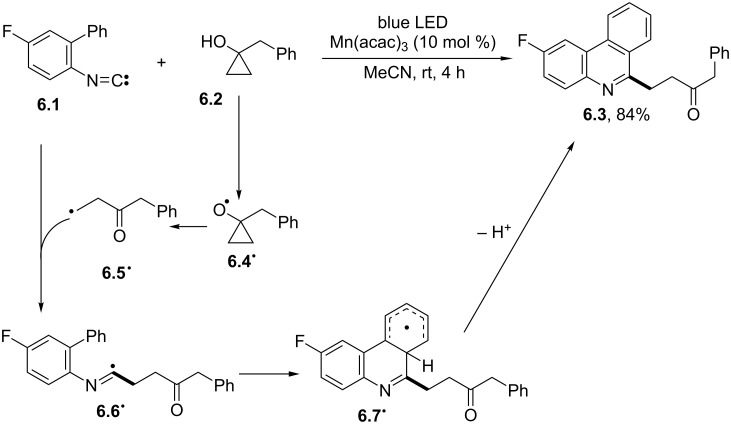
Photocatalyzed synthesis of 6-β-ketoalkyl phenanthridines.

A similar photocatalyzed tandem insertion/cyclization approach based on isocyanides and amino acid/peptide-derived Katritzky salts as precursors of *α*‐carbonyl radicals was likewise reported [50]. On the contrary, the Mn(acac)_3_ photocatalyzed ring opening of cyclopropanol **6.2** gave an easy access to a β‐carbonyl radical **6.5^·^**, which in turn added onto 2-biphenyl isocyanide **6.1** to give the corresponding 6-β-ketoalkyl phenanthridine **6.3** in a good yield (Scheme 6) [51].

**Scheme 7:**
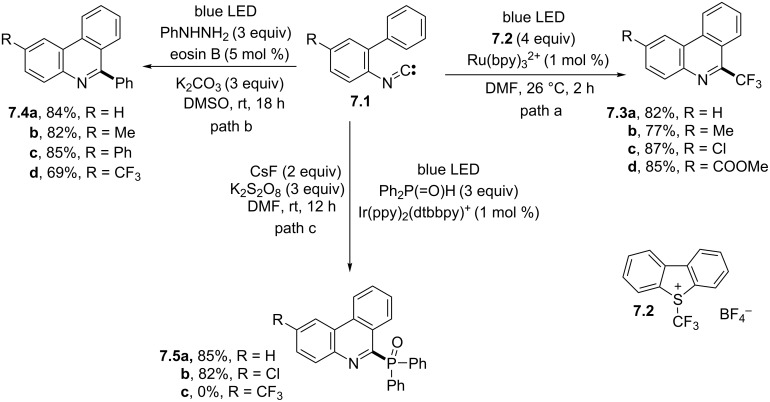
Synthesis of 6-substituted phenanthridines through the addition of trifluoromethyl (path a), phenyl (path b), and phosphonyl (path c) radicals to isonitriles.

The synthesis of perfluoroalkylated phenanthridines has been the subject of several studies in recent years. Accordingly, the use of perfluoroalkyl iodides and bromides for the synthesis of 6-trifluoroethyl [52], 6-difluoromethylphosphonated [53–54], and 6-mono- and difluoroalkyl- [55–56] phenanthridines was investigated. On the other hand, Umemoto’s reagent **7.2** was widely employed to introduce a trifluoromethyl group. In one instance, the visible-light irradiation of isocyanides **7.1** in the presence of excess **7.2** (4 equiv) and the Ru(bpy)_3_^2+^ photoredox catalyst afforded the desired trifluoromethylated products **7.3a–d** in satisfactory yields (Scheme 7, path a) [57]. Tri-, di-, and monofluoroalkylated derivatives were also obtained by using fluoroalkyl heteroaryl sulfones [58] or sodium sulfinates (in the presence of persulfate) [59] as the alkylating agents. In an alternative approach, sodium triflinate was adopted as the trifluoromethyl radical source along with diacetyl, that played the dual role of photomediator and reaction medium [60]. The same trifluoromethylated derivatives were obtained from **7.1** in the presence of CF_3_SO_2_Cl upon direct UV light irradiation by a Xe arc lamp (280–780 nm), in a photocatalyst-free fashion [61]. Easily scalable and thermally stable arylthiodifluoromethyl 2-pyridyl sulfones were likewise exploited in the visible-light photocatalyzed arylthiodifluoromethylation of differently substituted isocyanides [62].

6-Arylphenanthridines were obtained under photoredox-catalyzed conditions by using diaryldiodonium salts [57], arylsulfonyl chlorides [63], or aryl bromides [64] as the source of aryl radicals. A peculiar case is described in Scheme 7, path b, where arylhydrazines functioned as arylating agents to afford derivatives **7.4a–d** by having recourse to the photoorganocatalyst eosin B dye [65]. The generation of phenyl radicals from arylhydrazines was assured even when using the covalent organic framework 2D-COF-1 in place of eosin B [66]. Notably, the use of 2D-COF-1 allowed to extend the protocol to the synthesis of 6-alkylphenanthridines starting from alkylhydrazines [66].

However, a heteroatom-based radical may be used for the addition onto isonitriles as well. One such example dealt with the photoredox tandem phosphonylation/cyclization of diphenylphosphine oxides with 2-arylphenylisonitriles. Here, the sequential formation of C–P and C–C bonds gave P(=O)Ph_2_-containing phenanthridines **7.5a–c** (Scheme 7, path c), which occurred in the presence of a base (CsF or Cs_2_CO_3_) and an external oxidant (K_2_S_2_O_8_). Notably, the presence of electron-withdrawing groups on the biphenyl unit inhibited the process in some instances [67]. Starting from the same kind of substrates, 6-thiocyanatophenanthridines were isolated in discrete to excellent yields, in the presence of ammonium thiocyanate (NH_4_SCN) as the thiolating agent [68].

**Scheme 8:**
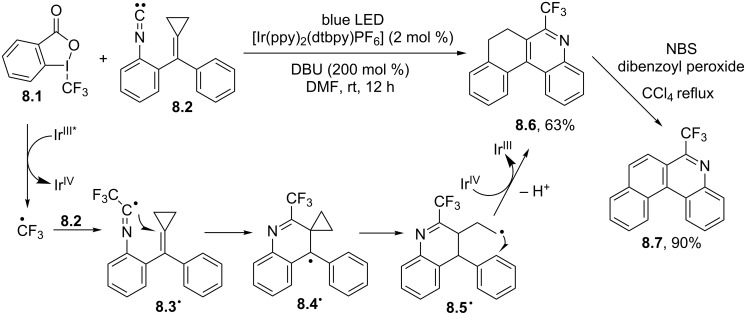
Synthesis of 6-(trifluoromethyl)-7,8-dihydrobenzo[*k*]phenanthridine.

A very peculiar case is that described in Scheme 8 for the synthesis of 6-(trifluoromethyl)-7,8-dihydrobenzo[*k*]phenanthridine **8.6** by the trifluoromethylation of methylenecyclopropane **8.2**. The reaction started with the generation of the trifluoromethyl radical via the Ir^III^ photocatalyzed reduction of Togni’s reagent **8.1**. The fluorinated radical added onto the isonitrile group present in **8.2** to give radical **8.3^·^**, which in turn gave intermediate **8.4^·^** upon cyclization onto the methylenecyclopropane double bond. Ring opening of the strained cyclopropyl ring liberated an alkyl radical (in intermediate **8.5^·^**) that readily cyclized onto the adjacent aromatic ring to give **8.6** in a good yield. The oxidation of **8.6** under radical conditions finally afforded the desired phenanthridine **8.7** in 90% yield [69].

**Scheme 9:**
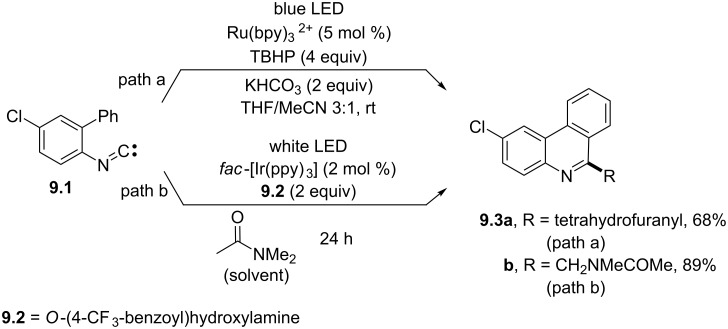
Phenanthridine syntheses by using photogenerated radicals formed through a C–H bond homolytic cleavage in THF (path a) and *N*,*N*-dimethylacetamide (path b).

Carbon-based radicals could be likewise generated via a C–H hydrogen-atom transfer path. As an example, ethers were used as hydrogen donors and underwent a C–H cleavage step promoted by a photogenerated *tert*-butoxyl radical. The so-obtained α-oxyalkyl radical intermediates were then trapped by biphenyl (or vinyl) isocyanides to afford functionalized phenanthridines, such as **9.3a** (or quinolines) (Scheme 9, path a) [70]. A photogenerated nitrogen-based radical was likewise used to cleave the C–H bond α-to-nitrogen in amides to form the corresponding α-amidoalkyl radicals for the synthesis of a set of 6-amidophenanthridines (e.g., **9.3b**) with significant antitumor and antimicrobial activities (Scheme 9, path b) [71].

**Scheme 10:**
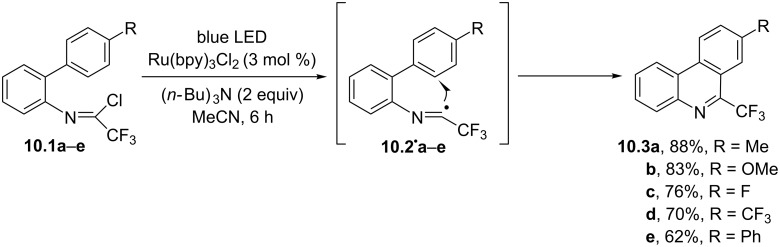
Trifluoroacetimidoyl chlorides as starting substrates for the synthesis of 6-(trifluoromethyl)phenanthridines **10.3a–e**.

Despite their extensive use, 2-isocyanobiphenyls or related isonitriles were not the only available substrates for the preparation of phenanthridines with the intermediacy of imidoyl radicals. As an example, the process depicted in Scheme 10 involved a visible-light homolytic radical aromatic substitution (HAS) starting from trifluoroacetimidoyl chlorides **10.1a**–**e**. Thus, the photocatalyzed cleavage of the C(sp^2^)–Cl bond in **10.1a–e** generated the corresponding imidoyl radicals **10.2^·^a–e** that, upon intramolecular radical cyclization, afforded 6-(trifluoromethyl)phenanthridines **10.3a**–**e** in very good yields [72].

**Scheme 11:**
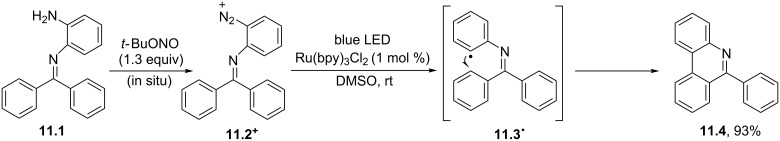
Synthesis of phenanthridines via aryl–aryl-bond formation.

A complementary approach in the synthesis of 6-arylphenanthridines started from *N*-(2-aminoaryl)benzoimine **11.1** and involved the formation of a C(sp^2^)–C(sp^2^) bond via an aryl radical intermediate (Scheme 11). Thus, compound **11.1** was in situ converted to the corresponding diazonium salt **11.2^+^**, which, upon reduction and nitrogen extrusion, formed the reactive aryl radical **11.3^·^**. In turn, the latter radical smoothly cyclized to form the desired phenanthridine **11.4** in excellent yield. Notably, the reaction could be readily applied to benzoimines having different substituents on the aromatic ring bearing the amino group [73].

**Scheme 12:**
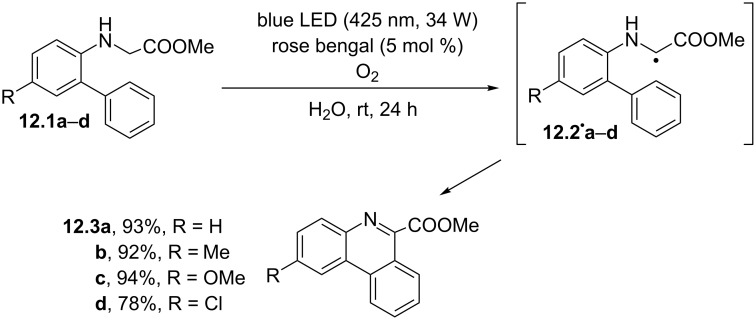
Oxidative conversion of *N*-biarylglycine esters to phenanthridine-6-carboxylates.

Glycine derivatives having a biaryl group attached to the *N*-terminus were successfully exploited for the construction of phenanthridine 6-carboxylates (Scheme 12). Notably, the process occurred in water under metal-free conditions in the presence of rose bengal (5 mol %) and made use of molecular oxygen as the terminal oxidant. Thus, *N*-biarylglycine esters **12.1a–d** promoted the reductive quenching of the excited photocatalyst, in turn triggering the formation of radicals **12.2^·^a–d**. These smoothly underwent radical cyclization to give the corresponding methyl 5,6-dihydrophenanthridine-6-carboxylates and then the desired phenanthridine 6-carboxylates **12.3a–d** in good yields. Noteworthy, the reaction could be scaled up to a 10 mmol amount, allowing to obtain grams of the desired phenanthridines, which could be isolated in a pure form by a simple filtration [74].

**Scheme 13:**
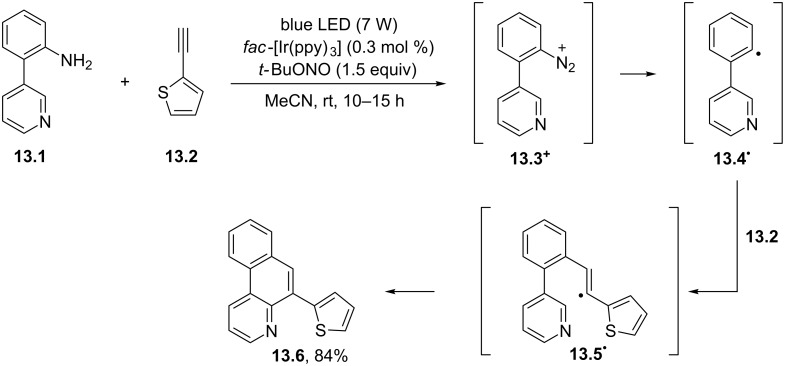
Photocatalytic synthesis of benzo[*f*]quinolines from 2-heteroaryl-substituted anilines and heteroarylalkynes.

Azaarenes different from phenanthridines (e.g., benzo[*f*]quinolines) could be likewise prepared by photocatalytic means. Thus, a highly regioselective strategy for the synthesis of a library of polyheteroaromatic compounds under photocatalytic conditions was reported (Scheme 13). The process made use of *fac*-Ir(ppy)_3_ (0.3 mol %) as the photoredox catalyst and occurred at room temperature under extremely mild conditions. The approach was based on the one-electron reduction of diazonium salts (see the case of **13.3^+^** in Scheme 13), formed in situ by the reaction of the chosen 2-heteroaryl aniline (e.g., **13.1**) with *tert*-butyl nitrite (1.5 equiv). Formation of the aryl radical **13.4^·^** and following addition onto an alkyne moiety (e.g., the 2-thienyl derivative **13.2**) afforded vinyl radical **13.5^·^**. The final intramolecular cyclization of **13.5^·^** and re-aromatization smoothly yielded the desired polyheteroaromatic derivative (see the case of **13.6**; 84% yield). Interestingly, all the obtained scaffolds bear two heteroatoms in close proximity to each other, prone to be engaged in a bidentate-type metal-coordination complex [75].

#### 2.2 Synthesis of phenanthridines via photocatalyzed C–N bond formation

**Scheme 14:**
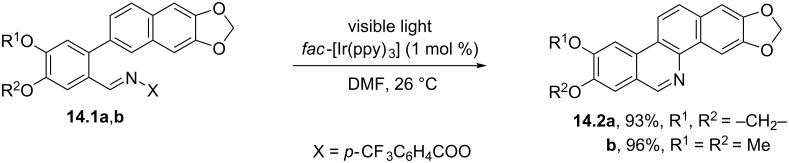
Synthesis of noravicine (**14.2a**) and nornitidine (**14.2b**) alkaloids.

As mentioned in the introduction, the examples gathered here involve the intermediacy of *N*-centered radicals. As a representative case, the photocatalyzed reduction of acyloximes **14.1a**,**b** offered a smooth entry to iminyl radicals (Scheme 14) [76]. The process took place at room temperature and involved the cleavage of a C–O bond, followed by a cyclization to give access to the benzo[*c*]phenanthridine alkaloids noravicine (**14.2a**) and nornitidine (**14.2b**) in almost quantitative yields [77].

**Scheme 15:**
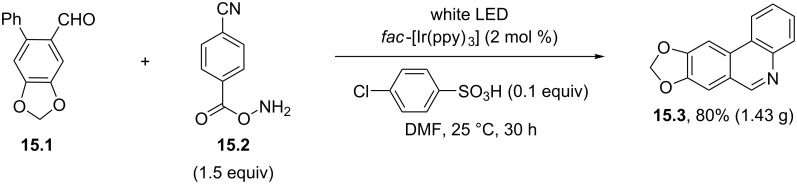
Gram-scale synthesis of the alkaloid trisphaeridine (**15.3**).

Acyloximes could be likewise formed in situ by the reaction of aldehydes with *O*-(4-cyanobenzoyl)hydroxylamine (**15.2**). The resulting adducts then underwent the same visible-light photocatalyzed cyclization with the intermediacy of iminyl radicals. Notably, the method was applied to the two-step synthesis of the alkaloid trisphaeridine (**15.3**) on a gram-scale quantity (Scheme 15) [78].

*O*-2,4-Dinitrophenyloximes were competent substrates for the photocatalyzed generation of iminyl radicals. In this case, the reaction was photoorganocatalyzed by eosin Y and took place in the presence of an excess (3 equiv) of a sacrificial donor, such as iPr_2_NEt [79]. Later, it was discovered that phenanthridines could be formed starting again from *O*-2,4-dinitrophenyloximes under photocatalyst-free conditions, by exploiting the capability of these oximes to form visible light absorbing EDA (electron donor–acceptor) complexes with Et_3_N. Thus, a good variety of highly functionalized phenanthridines was prepared in excellent yields [80].

**Scheme 16:**
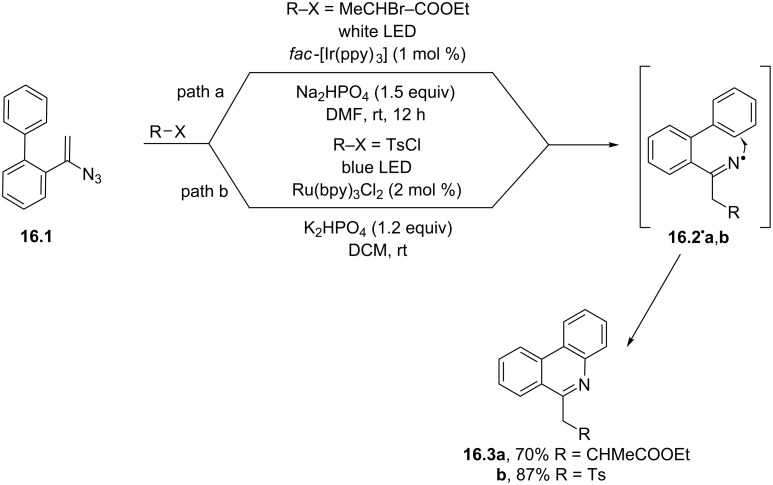
Synthesis of phenanthridines starting from vinyl azides.

Another approach for the visible-light-promoted generation of iminyl radicals (e.g., **16.2^·^a**,**b**) involved the addition of electrophilic radicals onto a vinyl azide (see the case of **16.1** in Scheme 16). Different radicals were used for this purpose. As an example, an α-carboxyethyl alkyl radical was formed from the corresponding α-bromoester under white LED irradiation in the presence of an Ir^III^-based photocatalyst. The addition of this intermediate onto the C–C double bond of **16.1** gave radical **16.2^·^a** upon nitrogen loss, which underwent an intramolecular cyclization and finally afforded the substituted phenanthridine **16.3a** in a satisfactory yield (Scheme 16, path a) [81]. The same azide **16.1** underwent trifluoromethyl radical addition to give the corresponding substituted phenanthridine. The F_3_C**^·^** radical was formed by the Fukuzumi catalyst Mes-Acr^+^ photocatalyzed oxidation of the Langlois reagent [82].

Sulfur-centered radicals may be generated via the reduction of sulfonyl chlorides and in turn exploited to construct 6-(sulfonylmethyl)phenanthridines via C–S bond formation. A typical case is shown in Scheme 16, path b. The process was initiated by the reduction of tosyl chloride (Ts–Cl) by a Ru^II^-based photocatalyst. The resulting sulfonyl radical afforded phenanthridine **16.3b** in a very good yield [83]. A related sulfonylation process was developed, starting from sulfonyl hydrazines in place of sulfonyl chlorides. In this case, the Ru^II^-based photocatalyst was able to reduce *tert*-butyl peroxybenzoate, triggering the release of a *tert*-butoxyl radical. This was in turn able to oxidize the hydrazine, allowing the liberation of the desired sulfonyl radical, prone to start a tandem sulfonylation/annulation of vinyl azides [84].

**Scheme 17:**
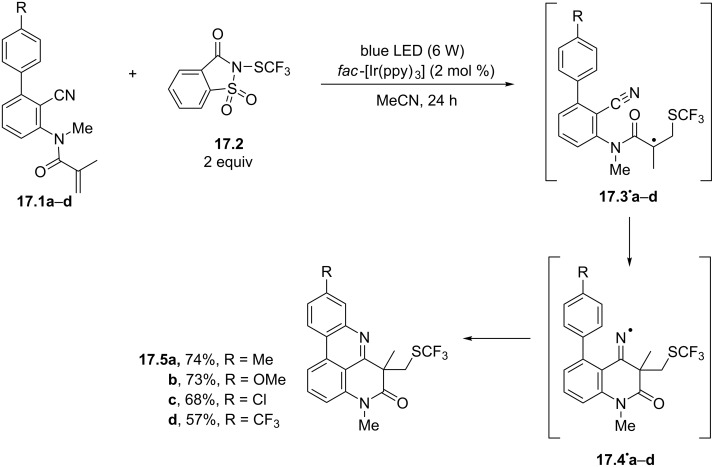
Synthesis of pyrido[4,3,2-*gh*]phenanthridines **17.5a–d** through the radical trifluoromethylthiolation of *N*-(*o*-cyanobiaryl)acrylamides **17.1a–d**.

Recently, the phenanthridine core was assembled through a radical cascade triggered by the trifluoromethylthiolation of *N*-(*o*-cyanobiaryl)acrylamides. The process occurred under visible-light irradiation (6 W blue LED) in the presence of the *fac*-Ir(ppy)_3_ photocatalyst (2 mol %). Among the tested sources of the CF_3_S**^·^** radical, *N*-(trifluoromethyl)thiosaccharin (**17.2**) offered the best performance (Scheme 17). Thus, the oxidative quenching of the excited Ir^III^-based photocatalyst allowed the generation of the desired (trifluoromethyl)thiyl radical, which added onto the double bond of **17.1a**–**d**, and finally delivered the desired products **17.5a**–**d** in good yields, through the intermediacy of radicals **17.3^·^a–d** and iminyl radicals **17.4^·^a–d** [85]. The double bond of acrylamides embedded into a 1,7-enyne framework likewise allowed the construction of the phenanthridone core by reaction with diethyl bromomalonate in the presence of *fac*-Ir(ppy)_3_. Notably, this process was characterized by mild conditions, operational simplicity, excellent functional group tolerance and offered high yields [86]. By following analogous approaches, the addition of perfluoroalkyl [87], acyloxy [88], or alkyl [89–90] radicals to the carbon–carbon double bond of the *N*-(*o*-cyanobiaryl)acrylamide skeleton led to the construction of differently substituted pyrido[4,3,2-*gh*]phenanthridines.

**Scheme 18:**
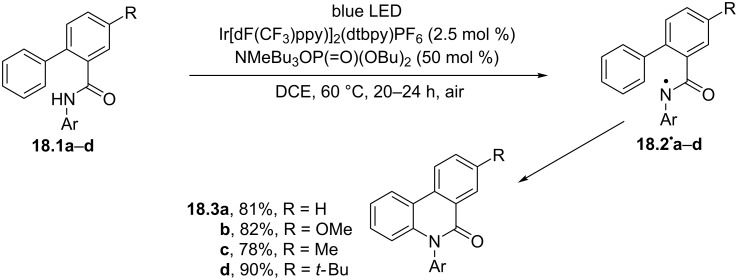
The direct oxidative C–H amidation involving amidyl radicals for the synthesis of phenanthridones.

Photocatalytically generated amidyl radicals were adopted for a direct oxidative C–H amidation, offering a straightforward access to phenanthridones (Scheme 18). The process took place upon blue LED irradiation (20–24 h at 60 °C were required) of the chosen substrates (e.g., **18.1a–d**) in the presence of the Ir-based photoredox catalyst Ir[dF(CF_3_)ppy]_2_(bpy)PF_6_ (2.5 mol %) and a phosphate base (50 mol %). Thus, the latter played a key role in the PCET event which triggered the activation of the N–H bond in **18.1a–d** and led to the *N*-centered radicals **18.2^·^a–d**. Ensuing cyclization onto the pendant aromatic group, followed by rearomatization enabled by molecular oxygen, gave the desired products **18.3a–d** in good yields [91]. Notably, a metal-free version of this strategy, based on the use of the 1-chloroanthraquinone photoorganocatalyst, was likewise reported [92]. A dual-catalytic system, comprising of eosin Y sodium salt (1 mol %) as photoredox catalyst and the thermal catalyst Pd(OAc)_2_ (5 mol %), was involved in the design of an efficient annulation between benzamides and in situ-generated arynes. The process occurred under oxygen saturated atmosphere at room temperature, likewise offering a straightforward access to the phenanthridone backbone [93].

## Conclusion

Photocatalysis is an important tool for the generation and exploitation of reactive intermediates in synthesis. The versatility of this approach allows to form in a straightforward manner several carbon and nitrogen-based radicals useful to forge C–C or C–N bonds (frequently, in an intramolecular fashion) for the construction of the tricyclic scaffold of phenanthrenes and their nitrogen-containing analogues, mainly phenanthridines. The adoption (in most cases) of visible light to promote the processes makes the photocatalytic approach one of the mildest methods available for the construction of these (hetero)aromatic rings. Most of the protocols illustrated herein, however, involved the use of rather expensive transition-metal-based (e.g., on Ru or Ir) photocatalysts, that still represents an issue in terms of sustainability. In this context, the use of photoorganocatalysts [24] is a promising opportunity on the route towards metal-free protocols for the synthesis of the phenanthrene and phenanthridine cores, a topic of current interest also in related thermal methods [94–95].
